# The Role of Endoscopy in the Diagnosis of Gastric Gastrointestinal Stromal Tumors

**DOI:** 10.1245/s10434-015-4520-5

**Published:** 2015-03-31

**Authors:** Toshirou Nishida

**Affiliations:** National Cancer Center Hospital East, Kashiwa, Chiba Japan

Gastrointestinal stromal tumor (GIST) is a rare cancer but the most common sarcoma in the gastrointestinal tract, arising most frequently in the stomach.^1^ Its clinical incidence is estimated to be 10 million/year. The standard treatment of primary and localized GIST is macroscopically complete resection. Gastric GIST shows significantly better prognosis after surgery than non-gastric GIST. GIST is considered to be originated from mesenchymal cells potentially differentiating into the interstitial cells of Cajal and mostly occurs in the intermuscular layer.^1^,^2^ Subsequent progression of GIST may be intraluminal, extrinsic, or bidirectional in the gastric wall. Practically, all tumors are covered by the normal mucosa and appear as submucosal (or subepithelial) tumors (SMTs) in endoscopic and fluoroscopic examinations. Hence, GIST is not directly visualized by endoscopy without using endoscopic ultrasound (EUS), and pathological specimens are not always obtained by conventional endoscopic biopsy. Because of its neoplastic behavior and localization, GIST may be sometimes asymptomatic until advanced stages and its histologic diagnosis prior to surgery is still challenging. The confronting problems include distinguishing the tumors with malignant behaviors and courses, which require treatment, from those with benign ones, which afford a watch-and-wait approach.

Endoscopy is of demonstrated value in diagnosis of esophageal and gastric carcinomas by identifying lesions and by providing tumor specimens for pathological examinations. In GIST and SMT, however, its diagnostic significance is not yet established. In this issue of the *Annals of Surgical Oncology*, Park et al. have shown some clear evidences for diagnostic roles of endoscopy and limitations of endoscopic examinations.^3^ Using the data obtained from their retrospective analysis of patients with histologically confirmed gastric GISTs, they shows that GISTs detected by periodical endoscopy are smaller in size, fewer in ulceration, and less symptomatic than those without periodical endoscopy when they show intraluminal growth, although endoscopy has a limited role in diagnosis of GISTs showing extrinsic growth. Alternatively, their data suggest that CT may work for extrinsic tumor and/or huge tumor >10 cm as an initial diagnostic approach. In fact, CT and MRI are proposed as a first choice to study location and extension of GIST in the guidelines.^4^ The other multicenter retrospective study has shown the alternative view that the patients with GISTs detected by gastric cancer screening without symptoms were smaller in size and better in prognosis than those with symptomatic GISTs.^5^ Taken together, periodical endoscopy may facilitate early detection of gastric GISTs before episodes, which may result in potential improvement in the prognosis of patients with gastric GISTs, although there may be lead-time bias.

This may evoke another important issue of whether all GISTs may require surgical resection when they are incidentally found by endoscopic screening. Pathological reports have shown unexpectedly high-frequency of small or microscopic GISTs in resection specimens for gastroesophageal malignancies.^1^,^2^ Although these small or microscopic GISTs may have similar histologic features and *KIT* mutations to clinical GISTs, they are shown to be practically indolent and may sometimes show involution in pathological examinations.^1^,^2^ Epidemiologic data also suggest that routine endoscopy found relatively-frequent neoplastic SMTs (nearly 0.1 %) in the stomach,^6^ half of which may be considered to be GISTs. NCCN and ESMO guidelines suggest that few small GISTs without high-risk EUS features, such as irregular border and heterogeneous internal echo, progress and become clinical GISTs, and suggest endoscopic follow-up for incidentally found-small GISTs lacking high-risk features until they grow or become symptomatic.^2^,^7^ GISTs increasing in size during periodical endoscopy, however, are shown to include a substantial number of intermediate or high-risk GISTs.^8^ Thus, indications of surgery for incidentally found GISTs and SMTs may include larger size, increase in size during follow-up, tumors with high-risk features, and symptomatic counterparts.^2^,^7^,^9^

The best frequency of endoscopic follow-up of asymptomatic GISTs remains uncertain. The GIST guidelines recommend annual follow-up by EUS for small GISTs.^2^,^7^,^9^ Increase in size is not commonly observed in small GISTs and some retrospective studies have indicated that <10–30 % of histologically proven GISTs increased during 2–6 years.^10^,^11^ The European Society of Gastrointestinal Endoscopy guidelines recommend triennial endoscopy for high-risk patients with Barrett’s esophagus, chronic atrophic gastritis, or intestinal metaplasia, who may develop esophageal or gastric carcinomas.^12^ Park et al. have reported that periodical endoscopy within 3 years did not increase the number of mitosis or high-risk GISTs, indicating that endoscopic or EUS follow-up every 3 years may work for small GISTs without high-risk features.^3^ Most of the results in this field, however, were obtained from retrospective cohort studies in single institutions. A prospective registry or randomized study is required to establish reliable evidences, even in rare disease, such as GIST.

In summary, endoscopy shows highly diagnostic performance in gastric GIST but has substantial limitations for tumors with extrinsic growth. When gastric GISTs (or SMTs) are found incidentally, EUS and/or CT may be recommended as an initial workup to evaluate whole pictures of tumors and high-risk features. Small GISTs lacking high-risk features could be followed by periodical endoscopy and/or EUS within 3 years.
